# Probiotic applications of bifidobacteria in poultry: administration methods and microencapsulation techniques

**DOI:** 10.20517/mrr.2025.64

**Published:** 2025-08-22

**Authors:** Eloy Argañaraz-Martínez, María Cristina Apella, Adriana Perez Chaia, Jaime Daniel Babot

**Affiliations:** ^1^Instituto de Microbiología, Facultad de Bioquímica, Química y Farmacia, Universidad Nacional de Tucumán, San Miguel de Tucumán T4000INI, Argentina.; ^2^Centro Científico Tecnológico NOA Sur, Consejo Nacional de Investigaciones Científicas y Técnicas (CONICET), San Miguel de Tucumán T4000, Argentina.; ^3^Laboratorio de Ecofisiología Tecnológica, Centro de Referencia para Lactobacilos (CERELA - CONICET - FML - FECIC), San Miguel de Tucumán T4000ILC, Argentina.

**Keywords:** *Bifidobacterium*, probiotic, poultry, gut health, microencapsulation, antibiotic alternatives

## Abstract

The search for sustainable alternatives to antibiotic growth promoters in poultry production has intensified in recent years, driven by global concerns over antimicrobial resistance and consumer demand for safer food systems. Among the probiotic candidates investigated, *Bifidobacterium* spp. stand out for their well-documented safety, immunomodulatory properties, and ability to enhance gut health. This review provides a comprehensive analysis of the biological roles, delivery strategies, and microencapsulation techniques for *Bifidobacterium* spp. as probiotics in poultry. Bifidobacteria contribute to poultry health by modulating the gut microbiota, improving intestinal morphology and digestive enzyme activity, and regulating immune responses through cytokine balance and epithelial barrier reinforcement. However, their strict anaerobic metabolism and sensitivity to gastric acid and processing conditions limit their viability during conventional administration. To address these challenges, we examine various administration routes, including oral, *in ovo*, spray/litter, and cloacal methods, highlighting their practical advantages and constraints. Special attention is given to microencapsulation technologies, such as spray drying, freeze drying, spray chilling, extrusion, and emulsion, which protect bifidobacteria from environmental stress and enhance their delivery to target intestinal sites. By integrating recent advances in biotechnology and delivery systems, this review underscores the potential of *Bifidobacterium* spp. as functional feed additives in antibiotic-free poultry production. Tailoring encapsulation materials and administration routes to match specific production goals is key to maximizing probiotic efficacy. Continued research on strain performance under commercial conditions will be essential to facilitate their large-scale application in sustainable poultry farming.

## INTRODUCTION

The poultry industry is a cornerstone of global food production, contributing significantly to protein supply and agricultural economies worldwide^[1]^. As demand for poultry meat and eggs continues to rise, particularly in low- and middle-income countries, maintaining the health and productivity of flocks has become more critical than ever. Traditionally, using antibiotics as growth promoters and disease prevention tools was a widespread and effective strategy^[2]^. However, mounting concerns about antimicrobial resistance, driven by the overuse and misuse of antibiotics in animal agriculture, have prompted regulatory restrictions and consumer-driven shifts toward more sustainable, antibiotic-free production systems^[3]^. In this context, the use of probiotics - defined as “live microorganisms that, when administered in adequate amounts, confer a health benefit on the host”^[4]^ - has emerged as a promising alternative to antibiotics in poultry farming. Among the diverse genera explored as probiotics, such as *Lactobacillus*, *Enterococcus*, *Streptococcus*, *Propionibacterium*, *Bacillus*, and *Saccharomyces*, *Bifidobacterium* stands out for its long-standing history of safe use in humans and animals, its extensive documentation in functional food applications, and its proven efficacy in modulating gut microbiota and host physiology^[5,6]^. Bifidobacteria are Gram-positive, non-motile, anaerobic microorganisms that naturally inhabit the gastrointestinal tract (GIT) of humans and animals, including poultry^[7]^, particularly in early life stages^[8]^. Their ability to adhere to the intestinal mucosa, produce organic acids, compete with pathogens, and modulate immune responses makes them suitable candidates for improving intestinal health and general well-being in poultry^[9]^. These attributes are especially valuable during critical periods such as early chick development, feed transitions, and stress or immunosuppression, when the intestinal microbiota is most vulnerable to disruption.

The safety of bifidobacteria is well established, with several species classified as GRAS (generally regarded as safe) by the Food and Drug Administration (FDA) and QPS (qualified presumption of safety) by the European Food Safety Authority (EFSA)^[10,11]^, supporting their use as probiotics in poultry production. While short-term trials (e.g., up to 42 days in Ross or Cobb broilers) consistently show that the administration of *Bifidobacterium* spp. improves performance and gut morphology with no adverse histological or immune effects^[12,13]^, comprehensive long-term safety data across full production cycles of laying hens remain limited. Multi-strain formulations containing *Bifidobacterium* spp. administered to broilers and layers have passed EFSA assessment for safety, with no observed negative effects on organ health or evidence of antimicrobial resistance gene transfer when used within recommended doses^[13-16]^. However, broader reviews caution that high-dose or prolonged probiotic use may, in rare cases, alter immune parameters, particularly cytokine expression or inflammatory markers [e.g., interleukin-6 (IL-6), IL-8], underlining the necessity of monitoring immune homeostasis over the full lifespan in diverse breeds^[17,18]^. No breed-specific histopathological abnormalities have been noted to date across quail, broilers, or laying hens.

Despite the promising potential of bifidobacteria, their application in poultry systems has been less explored than that of *Lactobacillus* and other species because they face substantial obstacles when implemented as probiotics in poultry production systems^[17]^. A primary challenge arises from their stringent anaerobic growth requirements. Unlike lactic acid bacteria (LAB), *Bacillus subtilis*, or yeast, bifidobacteria cannot tolerate oxygen exposure without significant loss of viability because they lack robust oxidative stress defense systems^[19]^. This sensitivity imposes strict conditions during industrial-scale fermentation, downstream processing, and handling stages, thereby increasing complexity and production costs. The inability to survive in the presence of oxygen also exponentially magnifies difficulties during feed formulation and storage, where oxygen exposure is inevitable. Consequently, bifidobacteria require specialized anaerobic fermenters and oxygen-impermeable packaging, driving up manufacturing expenses and limiting their widespread commercial use compared to more aerotolerant strains^[20]^. In addition to oxygen sensitivity, bifidobacteria generally have slower growth rates and lower biomass yields than *Bacillus* spp. and many LAB strains. This directly impacts production throughput and scalability, as achieving economically viable cell counts demands longer fermentation times and more controlled conditions^[20]^. *B. subtilis* provides a contrasting profile: it produces highly resistant endospores capable of surviving harsh physical and chemical stresses, including high temperatures during feed pelleting and acidic gastrointestinal environments^[21]^. These spores enable *Bacillus*-based probiotics to maintain functional viability throughout feed processing, storage, and gut transit, simplifying logistics and reducing losses in efficacy. Yeast probiotics, such as *Saccharomyces cerevisiae*, also demonstrate enhanced robustness under varying environmental conditions, including aerobic exposure and dehydration, adding to their practical advantages^[22]^. LAB, while more oxygen-tolerant than bifidobacteria, are not without their own drawbacks. Despite improved survival under oxygen exposure, many LAB strains exhibit limited thermal and moisture tolerances, which can adversely affect viability during conventional feed processing methods, such as pelleting processes that involve elevated temperatures and variable humidity^[23]^.

To overcome these challenges, recent advances in biotechnology have led to the development of microencapsulation techniques that protect probiotic bacteria during processing, storage, and passage through the harsh upper GIT. Microencapsulation involves the entrapment of live microorganisms within a matrix or coating material, such as alginate, chitosan, starch, or lipid-based systems, which shield the cells from environmental stressors and enable controlled release in the target intestinal site^[24]^. When tailored appropriately, microencapsulation not only enhances the viability of bifidobacteria but also improves their colonization efficiency and functional performance in the gut^[25]^.

The purpose of this review is to provide an updated and comprehensive overview of the probiotic applications of *Bifidobacterium* spp. in poultry production, with special emphasis on administration strategies and encapsulation technologies. We discuss the biological roles of bifidobacteria in poultry health, evaluate the current literature on their efficacy as dietary supplements, and examine the most recent innovations in encapsulation methods aimed at preserving their functional integrity. By bridging the gap between experimental findings and practical implementation, this review aims to support the integration of *Bifidobacterium*-based probiotics into sustainable poultry production systems.

### BIOLOGICAL ROLES OF BIFIDOBACTERIA IN POULTRY HEALTH

**Figure 1:**
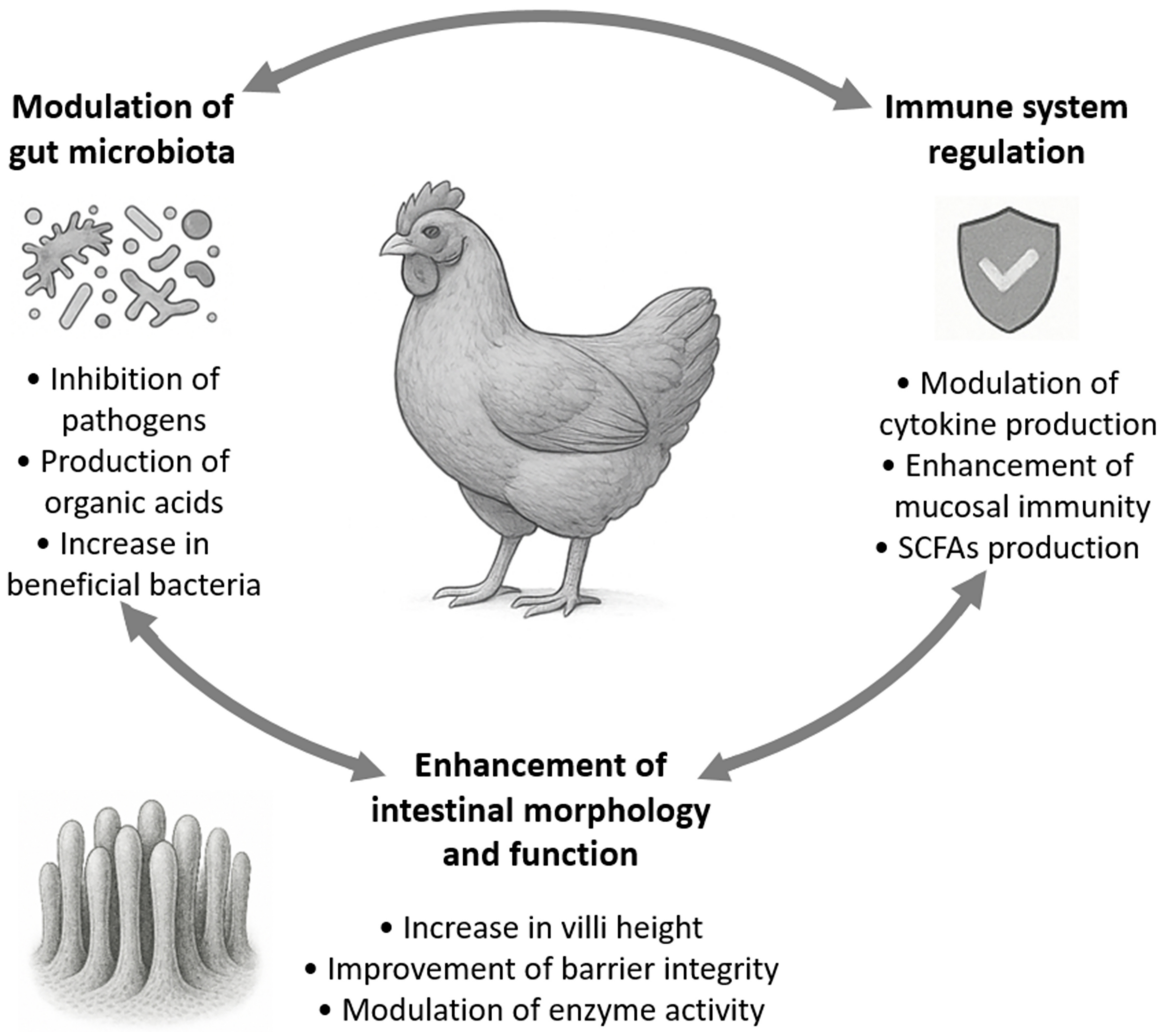
Interconnected probiotic functions of *Bifidobacterium* spp. in poultry. Arrows represent the synergistic relationships among the physiological benefits of the administration of bifidobacteria. SCFAs: Short-chain fatty acids.

Bifidobacteria have been increasingly recognized for their probiotic potential in poultry by improving the overall health of the birds^[26]^. As shown in Figure 1, their roles encompass modulation of gut microbiota, enhancement of intestinal morphology, immune system regulation, and protection against pathogenic infections. Altogether, these effects synergistically contribute to enhanced poultry productivity.

### Modulation of gut microbiota

The GIT of poultry hosts a complex and dynamic microbial community that plays a pivotal role in digestion, nutrient absorption, immune system development, and protection against pathogens. Among the beneficial microbes, bifidobacteria have garnered attention for their probiotic potential in modulating the gut microbiota to enhance poultry health and performance. Bifidobacteria can inhibit pathogenic bacteria through competitive exclusion, whereby they compete for adhesion sites and nutrients, effectively preventing colonization by harmful microbes. Additionally, bifidobacteria produce bacteriocins and organic acids like acetate and lactate, which lower the pH of the gut environment, creating unfavorable conditions for pathogenic bacteria^[27]^. In a recent study, Dixon *et al.* (2022) tested the inhibition of diverse poultry pathogens by *B. longum* ATCC 15708 *in vitro*^[28]^. This strain was able to inhibit the growth of common poultry pathogens, such as *Escherichia coli*, *Salmonella pullorum*, *Salmonella enterica* serovar Enteritidis, *S. enterica* serovar Typhimurium, *Enterococcus faecalis*, *Campylobacter coli*, *C. lari*, and *C. jejuni*, probably due to the production of organic acids. These authors proved the attachment of *B. longum* ATCC 15708 to intestinal mucosa from the duodenum of broilers, with rearrangement of the cell cytoskeleton that indicated a strong interaction, but the inhibition of pathogen adhesion was not tested. In another study, the inhibition of *Listeria monocytogenes* by this strain was attributed to the production of bacteriocins^[29]^. Kathayat *et al.* (2022) observed the presence of bioactive peptides in the culture supernatant of *B. lactis* Bb12, which were responsible for its bactericidal effect on *E. coli*^[30]^.

Besides inhibiting pathogenic bacteria, the supplementation with bifidobacteria has been shown to increase the abundance of beneficial microbial populations in the poultry gut. This shift in microbial composition contributes to improved gut health and function^[31]^, since gastrointestinal microorganisms hydrolyze dietary components to produce relevant metabolites^[32]^. For instance, dietary inclusion of a probiotic supplement composed of *E. faecium*, *Bifidobacterium*, and *Pediococcus acidilactici* (DSM Singapore Industrial Pte. Ltd.) enhanced the abundance of beneficial bacteria such as *Lactobacillus* and *Faecalibacterium*, while modulating the cecal microbiota structure of broilers^[31]^. The birds fed with the probiotic supplement presented an increased ratio of *Firmicutes* to *Bacteroidota*, which could improve the energy extraction from the feed. In another study, Liu *et al.* (2023) reported that the administration of a supplement composed of strains of *Bifidobacterium*, *L. casei*, and *L. acidophilus* improved the intestinal health of broilers by reducing the relative abundance of harmful bacteria, such as *Proteobacteria*, while increasing beneficial *Firmicutes*^[33]^. Furthermore, the introduction of bifidobacteria into the poultry diet has been linked to increased microbial diversity and stability within the gut ecosystem^[34]^. A diverse and stable microbiota is crucial for resilience against pathogenic invasions and for maintaining optimal digestive functions.

### Enhancement of intestinal morphology and function

The structural integrity and functional efficiency of the intestinal mucosa are critical for optimal nutrient absorption, immune defense, and overall health in poultry. Supplementation with bifidobacteria has been shown to positively influence intestinal morphology and function, leading to improved growth performance and disease resistance. A supplement containing *Bifidobacterium infantis* CRL1395, a strain with the ability to bind soybean agglutinin (SBA) in its surface, *Propionibacterium acidipropionici* LET 103, *Lactobacillus salivarius* LET 201, *L. reuteri* LET 210, and *E. faecium* LET 301 effectively protected the jejunal microvilli of broilers from damage and shortening caused by a diet rich in SBA^[35]^. Feeding *B. lactis* JYBR-190 to chicks infected with *S. pullorum* significantly increased duodenal and jejunal villus height and the ratio between villus height and crypt depth, indicating improved mucosal recovery and function^[36]^. These morphological changes, also observed by other authors on birds not challenged with pathogens^[37,38]^, expand the absorptive surface area, facilitating better nutrient uptake.

The intestinal barrier is essential for preventing pathogen translocation and maintaining gut homeostasis. The administration of a *Bifidobacterium* strain along with *Saccharomyces cerevisiae* Hansen and *Rhodopseudomonas sphaeroides* contributed to the reinforcement of this barrier by upregulating tight junction proteins such as zona occludens-1, claudin-1, and occludin in the jejunum of broilers^[38]^. Hu *et al.* (2024) observed increased expression of these proteins and junctional adhesion molecule-2 in the ileum of broilers fed with a combination of probiotics, including *Bifidobacterium* B8101, and betaine^[37]^.

The digestive efficiency of poultry is closely linked to the activity of intestinal enzymes such as amylase, lipase, and proteases. These enzymes are essential for the breakdown of macronutrients, facilitating nutrient absorption and overall growth performance. Recent studies have highlighted the role of bifidobacteria in modulating the activity of these enzymes, thereby enhancing digestive processes in poultry. The dietary inclusion of a probiotic mix including bifidobacteria significantly elevated the activities of amylase, lipase, trypsin, and chymotrypsin in the duodenum of broilers^[31]^. This enhancement in enzyme activities was associated with improved nutrient digestibility and growth performance, suggesting that bifidobacteria play a role in stimulating endogenous enzyme secretion or directly contributing exogenous enzymes to the digestive process.

### Immune system regulation

The immunomodulatory properties of *Bifidobacterium* spp. have been increasingly recognized in poultry production systems, where the enhancement of immune competence is a critical component of disease prevention and improved performance under intensive conditions. Supplementation with bifidobacteria has been shown to modulate both innate and adaptive immune responses, contributing to improved resistance to enteric infections and inflammatory challenges^[17]^. One of the primary mechanisms by which bifidobacteria exert their immunomodulatory effects is through the regulation of cytokine production. In a study by Yang *et al*. (2022), dietary administration of *B. lactis* JYBR-190 to chicks challenged with *S. pullorum* significantly downregulated the expression of pro-inflammatory cytokines such as tumor necrosis factor-alpha (TNF-α) and interferon-gamma (IFN-γ), while simultaneously upregulating the anti-inflammatory cytokine IL-10^[36]^. Moreover, bifidobacteria have been shown to enhance mucosal immunity by promoting the secretion of secretory immunoglobulin A (sIgA), which plays a central role in immune exclusion and protection against pathogenic colonization in the gut^[36]^. This immunoglobulin binds to antigens and microbial components, preventing their adherence and invasion of the intestinal epithelium, thereby contributing to a reinforced mucosal barrier.

Another important immunological effect of bifidobacteria pertains to the modulation of pattern recognition receptor pathways, particularly those involving Toll-like receptors (TLRs). In broiler models, probiotic supplementation has been associated with altered expression of TLR2 and TLR4, which are key in microbial recognition and the orchestration of downstream immune signaling^[33]^. By modulating these pathways, bifidobacteria may help balance immune activation and tolerance, mitigating excessive inflammatory damage. Furthermore, metabolites produced by bifidobacteria, particularly short-chain fatty acids (SCFAs) such as acetate and butyrate, play a significant role in modulating the host immune system in poultry. SCFAs have been shown to influence the differentiation and function of regulatory T cells (Tregs) through epigenetic mechanisms, including the inhibition of histone deacetylases and activation of G protein-coupled receptors such as GPR4 and GPR109A^[39]^. These metabolites also modulate cytokine production, enhancing anti-inflammatory mediators, such as IL-10, while suppressing pro-inflammatory cytokines, like TNF-α and IFN-γ^[40]^.

### EFFECT OF BIFIDOBACTERIA ON POULTRY PRODUCTIVITY

The administration of *Bifidobacterium* spp. has demonstrated promising effects not only on poultry gut health and immune modulation but also on key productivity parameters, such as body weight gain (BWG), feed conversion ratio (FCR), and carcass quality. These outcomes are especially relevant in the current trend of antibiotic-free production systems, where sustainable strategies to enhance performance are critically needed. Numerous studies have demonstrated that dietary inclusion of bifidobacteria or bifidobacteria-containing probiotic blends enhances growth performance in broilers by modulating the intestinal microbiota, specifically, increasing beneficial bacterial populations while suppressing pathogens, which directly contributes to improved FCR. For example, Wang *et al.* (2024) observed that a compound probiotic supplement including a *Bifidobacterium* strain (DSM Singapore Industrial Pte. Ltd.) significantly increased average daily gain (ADG) and body weight (BW) and reduced FCR by 7.0%, 6.9%, and 7.9%, respectively, in broilers over 42 days. These improvements were associated with increased SCFA concentration in the cecum (acetic acid, energy-supplying butyric acid, and total SCFA), better nutrient absorption, enhanced gut morphology (villus height and villus/crypt ratio in duodenum and jejune), and increased activity of digestive enzymes such as amylase, lipase, trypsin, and chymotrypsin in ileum and duodenum^[31]^. Liu *et al.* (2023) found that broilers receiving a probiotic mixture (basal diet + 1, 5, or 10 g of probiotic/kg) including a *Bifidobacterium* strain, *L. casei*, and *L. acidophilus* (Shanxi Ruimao Biotechnology Co., Ltd.) exhibited improved FCR and ADG compared to controls, especially for the higher dose of probiotic, which was attributed to enhanced fiber digestion and enzyme activities^[33]^. In another study, the 35-day administration of a supplement containing *B. animalis* subsp. *animalis* DSM 16284, *E. faecium* DSM 21913, and *L. salivarius* subsp. *salivarius* DSM 16351 (Biomin® C3, BIOMIN GmbH) led to a significant increase in the final BW of the birds at the minimum concentration of 1 × 10^8^ CFU/kg feed^[41]^. A synbiotic composed of *B. bifidum* and mannan oligosaccharide improved BWG and FCR, which was attributed to the modulation of the gut microbiota resulting from the production of antimicrobial substances by the bacteria, such as bacteriocins, hydrogen peroxide, and SCFAs, and the absorption of pathogenic bacteria by the prebiotic^[42]^. Combinations of mannan oligosaccharide (0.1% and 0.2%) and probiotic (1 × 10^6^ and 1 × 10^7^ CFU/g feed) were tested, with the most beneficial outcomes observed at 0.2% mannan oligosaccharide with 1 × 10^6^ CFU/g feed. In laying hens, supplementation with bifidobacteria has shown beneficial effects on laying performance and egg quality. Agustono *et al.* (2023) reported increased BW, feed intake, egg weight, height, and width, yolk index, albumin index, and morphology of the reproductive organs of laying hens administered with 1.2 to 7 × 10^9^ CFU/kg feed of *L. acidophilus*, *Bifidobacterium* spp., and *L. plantarum*, with the most pronounced effects observed at 7 × 10^9^ CFU/kg feed^[43]^. Nevertheless, the improvements in production parameters reported in these articles cannot be associated solely with *Bifidobacterium* spp. but with the feed supplements as a whole. Probiotic preparations composed solely of *Bifidobacterium* spp. strains have also been shown to exert beneficial effects on poultry productivity. Nour *et al.* (2021) informed that quails fed with a diet supplemented with 0.1% *B. bifidum* strain presented significantly higher BWG and better FCR than control birds, which could be due to improved digestive enzyme activities and inhibition of pathogens^[44]^. Similarly, the individual *in ovo* administration of 2 × 10^9^ CFU of *B. bifidum* ATCC 29521, *B. longum* ATCC 15707, *B. animalis* ATCC 27536, and *B. infantis* ATCC 15697 enhanced BWG and FCR of broilers after 28 days as a result of a combination of higher digestive enzyme activities, improved area for absorption of nutrients, and production of antimicrobial substances^[45]^. These changes were associated with an improved feed utilization produced by the probiotics. El-Moneim *et al.* (2020) reported improved FCR in broilers following *in ovo* administration of *B. bifidum* ATCC 29251 or *B. longum* ATCC 15707 at two different doses. However, hatchability was enhanced only in eggs injected with 5 × 10^9^ CFU of *B. bifidum* or 1 × 10^7^ CFU of *B. longum*, while BWG at day 35 improved in all treatment groups except those receiving *B. longum* at 5 × 10^9^ CFU^[46]^. El-Sharkawy *et al.* (2020) reported that broilers infected with *S.* Typhimurium and orally administered with 7 × 10^9^ CFU of *B. breve* JCM1192 or *B. infantis* BL2412 showed significantly higher BWG accompanied by enhanced gut microbial balance compared to infected controls^[47]^. In another study, FCR during the finisher period, live BW, and BWG of Japanese quails were improved by supplementing a basal diet with *B. bifidum* ATCC 29521 (5 × 10^8^ CFU/kg feed). These effects were attributed to reduced toxic compounds, modulation of the immune system, and nutrient digestion and absorption mediated by the probiotic strain^[12]^. Moreover, the overall growth and fertility were improved in male Japanese quails fed with a *B. bifidum* strain as a consequence of higher expression of estrogen receptors due to modulation of gut microbiota^[48]^.

Recent work demonstrates that the gut microbial baseline and probiotic responsiveness vary significantly across poultry breeds and species. For instance, Ross 308 broilers, Cobb 500 broilers, and Hy-line layer hens respond differently to the same probiotic blend: increases in villus height, crypt depth, and villus/crypt ratio are observed in Cobb 500 and layers, while Ross broilers often exhibit more modest histomorphometric changes and microbial modulation^[14]^. Likewise, native Indian chicken breeds exhibit distinctly different cecal microbiota composition compared to commercial broilers, suggesting breed-specific core microbiomes that may influence probiotic colonization and function^[49]^. These findings underscore that broiler-derived efficacy data cannot be directly extrapolated to layers, heritage breeds, or other poultry species like quail.

Specifically, studies in Japanese quail supplemented with *B. bifidum* ATCC 29521 (alone or combined with *B. toyonensis* ATCC 55050) reported improvements in feed efficiency, egg weight, fertility, and hatchability, but with a smaller magnitude in FCR and weight gain than seen in broilers^[50]^. This difference may reflect the unique gut physiology and slower growth kinetics of quails. In laying hens, blends containing *Bifidobacterium* spp. and *L. casei* strains improved egg weight and yield, but these effects varied by strain and duration, a contrast to more robust broiler growth responses^[15]^.

Overall, the integration of *Bifidobacterium* spp. into poultry nutrition programs can positively impact productivity through multiple mechanisms, including microbiota modulation, improved nutrient digestibility, enhanced intestinal structure, and reduced pathogen pressure. *Bifidobacterium* spp. modulate the intestinal microbiota of broilers, increasing the proportion of *Firmicutes* to *Bacteroidota*, which results in increased energy extraction from dietary fiber, positively affecting BW^[51]^. Amylase, lipase, trypsin, chymotrypsin, and other enzymes with increased activities in the gut of birds administered with *Bifidobacterium* spp. play a crucial role in the hydrolysis, digestion, and absorption of crude proteins, lipids, and carbohydrates, converting them into amino acids, triglycerides, and glucose in broilers. Consequently, the enhanced activity of digestive enzymes observed with the probiotic supplementation may account for improved nutrient digestibility and growth performance^[31]^. This elevation in enzymatic activity is thought to result either from direct secretion of digestive enzymes by probiotics, stimulation of host cell secretion, or a synergistic effect of both mechanisms, ultimately facilitating greater digestive enzyme production^[52]^. *Bifidobacterium* spp. have been shown to increase SCFA in the cecum, which supports the maintenance of intestinal mucosa integrity, while some SCFAs can also be directly absorbed as nutrients^[53]^. In addition, SCFAs lower the intestinal pH, inhibiting pathogens^[31]^. Moreover, broilers supplemented with *Bifidobacterium* spp. exhibited improved intestinal morphology, characterized by longer villi and a higher villus/crypt ratio. These structural enhancements are likely to facilitate more efficient nutrient absorption, thereby contributing to improved growth performance^[36]^. These effects are highly strain-specific and dose-dependent, necessitating careful selection and standardization for optimal performance outcomes^[54]^. Continued research under commercial-scale conditions is essential to validate these benefits and to fine-tune formulation strategies that maximize productivity while maintaining animal welfare and sustainability.

To translate these benefits into practical outcomes, it is crucial to evaluate the different methods of administering *Bifidobacterium* spp. in poultry systems.

### ADMINISTRATION ROUTES OF BIFIDOBACTERIA IN POULTRY

The successful application of *Bifidobacterium* strains as probiotics in poultry production relies not only on strain selection and viability but also on the method of administration. The route by which bifidobacteria are delivered influences their capacity to colonize the GIT, survive the host’s physiological barriers, and exert their intended immunological and metabolic effects. Effective colonization is a prerequisite for the modulation of host-microbe interactions, including competitive exclusion of pathogens, enhancement of mucosal immunity, and production of bioactive metabolites such as SCFAs^[36]^. Consequently, selecting an appropriate administration route is pivotal for optimizing the probiotic’s efficacy in poultry systems.

Several delivery strategies have been developed and tested to ensure that probiotic bifidobacteria reach their target sites in the gut in sufficient numbers and remain viable during the course of intestinal transit. These include *in ovo* injection, oral supplementation via feed or water, spray application onto feathers or litter, and more invasive routes such as cloacal or direct gastrointestinal delivery^[47,55-57]^. Each method offers distinct advantages in terms of early microbial programming, logistical feasibility, and compatibility with large-scale poultry operations. However, they also present unique limitations that may influence colonization kinetics, probiotic stability, and overall performance outcomes. On the other hand, authors suggested that combinations of administration routes are more efficient to prevent pathogen colonization^[58,59]^. The main advantages and disadvantages of each administration route are summarized in Table 1.

**Table 1:** Comparative analysis of *Bifidobacterium* administration routes in poultry production

	**Administration route**			
	**Oral administration**	***In ovo* injection**	**Spray and litter application**	**Cloacal and direct gastrointestinal administration**
**Advantages**	- Simple, non-invasive, and scalable - Easily incorporated into feed or water - Allows repeated dosing	- Enables colonization before hatch - Promotes early immune system development - Standardizable via automated systems	- Mass application without handling birds - Ingestion via preening or environment - Useful in hatcheries and brooding areas	- Bypasses acidic and oxygenated upper GIT - Directly delivers bacteria to their ecological niche - Promotes fast and stable colonization at the ceca
**Disadvantages**	- Exposure to oxygen during storage and handling - Harsh gastric conditions reduce viability - Often requires protective encapsulation	- Requires technical precision - Risk of embryonic mortality - Limited probiotic dosage volume - Exposure to oxygen during formulation and injection	- High oxygen exposure reduces anaerobe viability - Inconsistent individual exposure - Dependent on bird behavior and environment	- Impractical for mass application - Labor-intensive with high handling stress - Potential risk of cross-contamination between birds
**Anaerobic suitability**	Low	Low-Moderate	Low	High
**Early microbial programming**	Moderate-high	Very high	Moderate	High
**Colonization efficiency**	Moderate	High	Variable	Very high
**Microencapsulation requirement**	High	High	High	Low
**Logistical feasibility**	Very high	Moderate	Very high	Very low
**Large-scale compatibility**	Very high	High	Very high	Very low

GIT: Gastrointestinal tract.

As our understanding of host-microbiota interactions deepens, it becomes increasingly clear that the timing, dosage, and route of administration must be tailored to specific production goals, whether to support early-life immune development, improve feed conversion, or enhance resistance to enteric pathogens^[34]^. Thus, optimizing delivery methods is not merely a technical issue but a strategic one that integrates microbiological, immunological, and zootechnical knowledge.

### Oral administration

Oral administration represents the most prevalent and operationally feasible method for delivering *Bifidobacterium* spp. in commercial poultry systems, primarily through incorporation into feed or drinking water^[60]^. This route is applicable across diverse poultry categories, including broilers, layers, and breeders, with particular emphasis on neonatal and juvenile stages due to their heightened responsiveness to microbial modulation^[61]^. Delivery via water offers flexibility in dosage and timing, promoting consistent exposure during critical early-life periods, and is often more effective than feed-based delivery, especially in newly hatched chicks that may not readily access solid feed in the first 24-48 h^[62,63]^. Early oral supplementation, beginning immediately post-hatch, plays a pivotal role in microbial programming of the GIT. Establishing beneficial microbial populations early in life not only shapes a stable gut microbiota but also promotes intestinal maturation and primes the host immune system, thereby enhancing resilience to pathogens^[64]^. From a logistical standpoint, oral administration integrates seamlessly with existing infrastructure, such as automated feeding and watering systems, enabling uniform delivery to large flocks with minimal additional labor or cost. In broilers, whose production cycles are rapid and tightly synchronized, consistent and scalable delivery is particularly crucial. Similarly, in layer and breeder operations, the maintenance of long-term intestinal health and productivity benefits from periodic supplementation during stress-associated periods such as vaccination or peak laying phases^[65]^. However, several limitations must be acknowledged. The survivability of *Bifidobacterium* strains through the upper GIT and during feed processing (e.g., pelleting) is often compromised due to exposure to oxygen, heat, pressure, and moisture^[66]^. Furthermore, environmental factors such as water chlorination, pH, and storage conditions can negatively impact probiotic viability^[67]^. These stability issues can lead to inconsistent colonization kinetics, ultimately influencing the efficacy of the probiotic intervention. Although numerous studies have reported improvements in performance parameters, such as FCR, growth rate, and reduced morbidity, these outcomes are highly dependent on strain selection, dosage, and timing of administration^[64]^. Therefore, to fully leverage the benefits of oral administration, it is imperative to optimize delivery protocols through protective formulation strategies (e.g., microencapsulation), careful strain selection, and standardized dosing regimens tailored to the physiological and immunological needs of specific poultry types and developmental stages.

### *In ovo* injection

*In ovo* injection of *Bifidobacterium* spp. presents a strategic and increasingly explored route for early microbial programming in poultry, whereby probiotics are delivered into the amniotic sac during late embryogenesis (typically day 17-18 of incubation)^[68]^. This approach is predominantly applied in broiler chickens due to the tight synchronization of their incubation and hatching processes, and the high scalability of commercial hatchery systems^[69]^. *In ovo* delivery enables the colonization of the GIT with beneficial bacteria before hatch, which can suppress early colonization by opportunistic and pathogenic microbes, stimulate mucosal immunity, and support intestinal maturation. Furthermore, in fast-growing broilers with a short production cycle, early-life microbial modulation can yield measurable improvements in performance and health outcomes within a few weeks^[34]^. Studies have demonstrated that *in ovo* application of *B. saeculare* B2-2 and B3-4, either alone or combined with LAB, reduces post-hatch colonization by gram-negative bacteria and *Enterococcus* spp., while enhancing early BWG and intestinal morphology^[56]^. While most evidence comes from broiler models, less is known about its utility in layer-type chickens, where embryonic development timelines and commercial incubation practices differ slightly and may require adjusted protocols^[70]^. The *in ovo* administration of probiotics in poultry species other than chickens remains poorly investigated and, to date, has yielded limited or inconsistent results^[71]^. From a practical standpoint, this technique is compatible with existing automated vaccine delivery systems in commercial hatcheries, offering a logistically feasible and scalable solution for mass administration^[72]^. However, challenges remain regarding probiotic strain stability, survival during *in ovo* delivery, and standardization of dosing without compromising hatchability^[73]^. Moreover, while early benefits are clear, sustained colonization and long-term functional outcomes may require subsequent reinforcement through post-hatch supplementation^[74]^. Despite these limitations, *in ovo* injection offers a promising route to establish health-promoting microbiota during a critical developmental window, aligning microbiological efficacy with the practical demands of modern poultry production systems.

### Spray and litter application

Spray and litter application of probiotics has emerged as a non-invasive and operationally practical strategy for probiotic delivery in poultry, particularly during the early post-hatch period^[58]^. This method involves spraying probiotic suspensions directly onto eggshells, newly hatched chicks, hatchery trays, or litter substrates, thereby enabling rapid exposure and oral ingestion of beneficial microbes through preening and environmental contact^[75]^. It is especially suited for neonatal chicks in broiler and layer operations, when the immune system and GIT are still under development and most susceptible to microbial programming^[76]^. In broilers, early colonization via this method can promote intestinal barrier maturation and reduce early-life susceptibility to enteric infections^[76]^. In layer-type chicks, litter application during brooding can aid in establishing a stable gut microbiota, with potential long-term effects on productivity and health during the laying cycle. The appeal of this method lies in its simplicity, compatibility with existing hatchery and barn equipment, and potential to uniformly treat large bird populations without individual handling^[77,78]^. Furthermore, this method minimizes labor and stress associated with oral gavage or feed incorporation, making it logistically advantageous in commercial operations. Early exposure via this route may help shape the gut microbial ecosystem during critical developmental windows, promoting intestinal maturation and immune priming^[58]^. However, the application of strictly anaerobic species such as *Bifidobacterium* spp. via spray or litter raises specific challenges related to their oxygen sensitivity, which may be overcome through protective technologies such as microencapsulation. Bifidobacteria are highly susceptible to oxidative stress, which can significantly reduce their viability during preparation, storage, and environmental exposure^[79]^. Upon aerosolization or contact with ambient air, the metabolic activity and survival of these organisms may be compromised, undermining colonization efficiency and functional efficacy. Furthermore, the uneven distribution of probiotics in the litter or on chick surfaces can lead to inconsistent intake among individuals, reducing the uniformity of microbiota establishment. These issues are more pronounced in older birds, such as growers and adults, where behavioral patterns and immune status differ, and this route of administration is less commonly used due to reduced environmental susceptibility and lower interaction with litter surfaces.

### Cloacal and direct gastrointestinal administration

Cloacal and direct gastrointestinal administration of *Bifidobacterium* spp., though less conventional than oral or *in ovo* routes, offer targeted delivery directly to the lower intestine, bypassing the hostile upper GIT conditions (i.e., low pH and proteolytic enzymes) and minimizing loss of probiotic viability through gastric digestion^[80]^. In this approach, probiotics are delivered either via cloacal inoculation or direct deposition into specific gut segments, ensuring immediate access to the ceca and colon, which are critical sites for microbial interaction and metabolic activity^[81,82]^. These routes are primarily applied in neonatal chicks or juvenile poultry under experimental or controlled production settings, where early colonization can influence the trajectory of microbiota establishment and immune development^[80]^. In the case of cloacal administration, the procedure typically involves the use of a sterile, blunt-end polyethylene or polypropylene pipette or cannula, carefully inserted approximately 1.5-2.0 cm into the cloaca of day-old chicks, depending on body size, to avoid injury or perforation of the rectal mucosa^[83]^. The volume of inoculum generally ranges from 50 to 100 µL per bird and must be delivered slowly to prevent expulsion. Aseptic conditions are critical throughout the process to avoid introducing opportunistic pathogens; thus, sterile gloves, disinfected equipment, and a clean environment are essential. Proper restraint of the bird is also important to minimize stress and ensure accurate deposition. Unlike oral or spray routes, which expose bacteria to atmospheric oxygen, this method delivers the probiotic directly into the lower GIT, which is more anaerobic, enhancing the survival and functional activity of strict anaerobes, such as *Bifidobacterium* spp. In day-old chicks, whose lower GIT is still maturing and relatively oxygen-rich, direct inoculation can facilitate the early dominance of beneficial anaerobes before competitive exclusion by endogenous flora is fully established^[84]^. However, the use of this method in adult birds is rare due to practical limitations associated with bird size, handling complexity, and reduced responsiveness of the mature GUT to microbial manipulation. While these routes excel in ensuring probiotic survival and immediate local effects on the mucosal immune system and microbial communities, practical constraints limit their scalability under commercial conditions. Individual handling of birds is labor-intensive and time-consuming, and it carries risks of cross-contamination and bird stress, which may impact welfare and performance outcomes^[85]^. Additionally, the long-term residence and functional impact of the introduced bacteria depend on continued competitive activity and may require subsequent supplementation to maintain colonization and performance benefits^[85]^. Therefore, although cloacal and direct gastrointestinal delivery methods provide precise and potent probiotic deployment and are valuable for research and targeted interventions, their integration into large-scale operations necessitates simplified administration techniques or automation to balance efficacy and practicality.

While the choice of administration route determines the extent of probiotic colonization and functional activity, the success of any method ultimately depends on the ability of bifidobacteria to survive processing, storage, and the physiological challenges of the GIT. This need for enhanced stability has driven increasing attention toward microencapsulation technologies, which provide protective barriers and controlled delivery systems.

### MICROENCAPSULATION TECHNIQUES FOR BIFIDOBACTERIA

Microencapsulation has emerged as a pivotal strategy to protect oxygen-sensitive *Bifidobacterium* spp. during industrial processing, storage, and passage through the harsh environment of the GIT^[86]^. The encapsulation of probiotic cells within biopolymeric matrices forms a physical barrier that preserves cell viability amid adverse conditions, such as high temperatures, oxygen exposure, and acidic pH^[87]^. The addition of antioxidants within the encapsulation matrix can help mitigate this issue to some extent^[88]^. Literature in food biotechnology emphasizes that successful microencapsulation enhances functional probiotic performance in food and feed applications by stabilizing cell membrane integrity and facilitating controlled release at the target site within the host GIT^[89-91]^. Microencapsulation plays a crucial role not only in preserving the viability of bifidobacteria but also in maintaining their surface structures, which may undergo alterations prior to reaching their target site of action. The bioactive molecules mediating interactions between the microorganism and the host predominantly reside on the bacterial cell surface^[92]^. Key components include exopolysaccharides (EPSs), cell wall polysaccharides, (lipo)teichoic acids (LTAs), glycolipids, peptidoglycan, and surface proteins. Variations in EPS structure influence the adhesion capacity of *Bifidobacterium* strains to intestinal epithelial cell lines, as well as modulating peripheral blood mononuclear cell proliferation and cytokine secretion *in vitro*^[93]^. Moreover, evidence suggests that neutral EPSs and those with high molecular weight tend to suppress pro-inflammatory cytokine production, whereas low molecular weight or acidic EPSs exhibit immunostimulatory effects^[93]^. Cell wall polysaccharides contribute to pathogen control by obstructing potential binding sites on the intestinal epithelium^[94]^, enhance bacterial resilience under environmental stress, and promote biofilm formation within the intestine^[95]^. Recognition of LTAs is mediated via the TLR2/TLR6 heterodimer complex, which involves co-receptors CD14 and CD36^[96]^. The glycolipids on the surface of bifidobacteria are detected by host pattern recognition receptors TLR2, TLR1, and TLR6. Notably, TLR2 is pivotal for recognition of several bacterial surface antigens such as lipids, LTAs, and potentially peptidoglycan; however, the recognition of lipoproteins and lipopeptides requires TLR2 to form heterodimers with either TLR1 or TLR6^[97,98]^. The peptidoglycan of *B. breve* YY induces differentiation of naïve T cells toward Th1 and promotes dendritic cell maturation^[99]^. Furthermore, studies employing the RAW 264.7 macrophage cell line have revealed that cell wall extracts from bifidobacteria stimulate production of TNF-α, IL-6, and nitric oxide^[100-102]^. Among surface proteins, serpin and pilin are notable; serpin functions primarily by inhibiting host or microbial proteases, thereby enhancing the survival and colonization capacity of bifidobacteria within the GIT^[103]^. Effective colonization of the gut by bifidobacteria is also dependent on pili formation, mediated by pilin proteins, which facilitate bacterial adhesion to the mucosal surfaces of the host intestine^[104]^.

Advances in encapsulation technologies, including spray drying, freeze drying, spray chilling, extrusion, and emulsion-based methods, offer modular platforms to adjust capsule size, release kinetics, and carrier composition, thereby tailoring probiotic delivery to specific production needs^[105]^. Strategic selection of wall materials such as polysaccharides (e.g., alginate, gum Arabic, *etc*.), proteins (e.g., whey, soy, *etc*.), and prebiotic blends (defined as a substrate that is selectively utilized by host microorganisms to confer a health benefit^[106]^; e.g., inulin conjugates, among others) further strengthens bacterial survival under aerobic storage and digestive stress, ultimately improving population levels at the site of action^[107]^. As such, microencapsulation stands as a cornerstone technology that bridges microbiological efficacy with logistical practicality, enabling the effective use of *Bifidobacterium* spp. as functional feed additives in poultry production. Selected recent reports on the microencapsulation of *Bifidobacterium* spp. are detailed in Table 2.

**Table 2:** Selected recent reports on the microencapsulation of *Bifidobacterium* spp.

**Method**	**Strain**	**Encapsulating matrix**	**Encapsulation efficiency (%)**	**Particle size (µm)**	**Viability during storage**	**Resistance to gastrointestinal digestion**	**Key parameters**	**Reference**
Spray drying	*B. lactis* B94	Polyvinylpyrrolidone polymer and lactose	99.14^*^	ND	Decrease from 9.18 ± 0.04 to 8.19 ± 0.02 log CFU/g (33 days at 25 ºC)	ND	IAT: NI; OAT: NI	[108]
Spray drying	*B. animalis* subsp. *lactis* BB-12	Goat’s milk	96.97^*^	ND	ND	ND	IAT: 150 ºC; OAT: 50 ± 3 ºC	[109]
Spray drying	*B. animalis* subsp. *lactis* BB-12	Goat’s milk and inulin	92.58^*^	ND	ND	ND	IAT: 150 ºC; OAT: 50 ± 3 ºC	[109]
Spray drying	*B. animalis* subsp. *lactis* BB-12	Goat’s milk and oligofructose	90.49^*^	ND	ND	ND	IAT: 150 ºC; OAT: 50 ± 3 ºC	[109]
Spray drying	*B. animalis* subsp. *lactis* BB-12	Goat’s milk, inulin, and oligofructose	86.12^*^	ND	ND	ND	IAT: 150 ºC; OAT: 50 ± 3 ºC	[109]
Spray drying	*B. bifidum*	Gum arabic and β-cyclodextrin	ND	ND	Decrease from 5.7 ± 0.2 to < 1.0 log CFU/g (90 days)	ND	IAT: 120 ºC; OAT: 50 ºC	[110]
Spray drying/spray chilling	*B. bifidum*	Gum arabic, β-cyclodextrin, hydrogenated palm oil, and Tween 80	ND	ND	Decrease from 3.5 ± 0.2 to < 1.0 log CFU/g (90 days)	ND	IAT: 120 ºC; OAT: 50 ºC (spray drying) Feeding temperature: 45 ºC; Nozzle temperature: 38 ºC (spray chilling)	[110]
Freeze drying/nanoemulsion	*B. bifidum* NRRL B-41410	Clay hydrophilic bentonite, whey protein concentrate, sodium alginate, and maltodextrin	88.84	ND	Increase from 7.33 to 8.27 log CFU/g (20 days at 7 ºC)	69.23% survival (sequential exposure to gastric and intestinal juices)	Vacuum degree: NI	[111]
Freeze drying/nanocomposite	*B. bifidum* NRRL B-41410	Clay hydrophilic bentonite, whey protein concentrate, sodium alginate, and maltodextrin	98.49	ND	Increase from 7.48 to 8.40 log CFU/g (20 days at 7 ºC)	68.84% survival (sequential exposure to gastric and intestinal juices)	Vacuum degree: NI	[111]
Spray chilling	*B. bifidum*	Hydrogenated palm oil and Tween 80	ND	ND	Decrease from 6.1 ± 0.1 to 2.4 ± 0.1 log CFU/g (90 days)	ND	Feeding temperature: 45 ºC; Nozzle temperature: 38 ºC (spray chilling)	[110]
Spray chilling/spray drying	*B. bifidum*	Hydrogenated palm oil, tween 80, gum arabic, β-cyclodextrin, and lecithin	ND	ND	Decrease from 3.6 ± 0.1 to 2.3 ± 0.2 log CFU/g (90 days)	ND	Feeding temperature: 45 ºC; Nozzle temperature: 38 ºC (spray chilling) IAT: 120 ºC; OAT: 50 ºC (spray drying)	[110]
Extrusion	*B. lactis*	Alginate, hydroxypropyl methyl cellulose, gellan gum, carboxymethyl chitosan with polyethylenimine	ND	ND	Decrease of 1.64 ± 0.17 log CFU/g (12 weeks at 4 ºC) Decrease of 2.91 ± 0.23 log CFU/g (12 weeks at 30 ºC)	Decrease of 0.59 ± 0.02 (exposure to simulated gastric fluid) and 1.19 ± 0.05 log CFU/g (exposure to simulated intestinal fluid)	Frequency: 300 Hz; Voltage: 500 V; Nozzle aperture: 750 µm	[90]
Extrusion	*B. pseudocatenulatum* G7	Sodium alginate	ND	462	Decrease from ~8.06 to ~5.74 log CFU/g (4 weeks at 4 ºC)^**^	Decrease from ~9.18 to ~4.58 log CFU/g (exposure to simulated gastric fluid); no detectable after complete gastrointestinal digestion^**^	Frequency: 800 Hz; Voltage: 800 V; Nozzle aperture: 200 µm	[112]
Extrusion	*B. pseudocatenulatum* G7	Sodium alginate and CaCO_3_	ND	520	Decrease from ~8.73 to ~6.93 log CFU/g (4 weeks at 4 ºC)^**^	Decrease from ~6.44 to ~8.70 (exposure to simulated gastric fluid), and to ~5.60 log CFU/g (complete gastrointestinal digestion)^**^	Frequency: 800 Hz; Voltage: 800 V; Nozzle aperture: 200 µm	[112]
Extrusion	*B. animalis* subsp. *animalis* ATCC 25527	Ciceritol and sodium alginate	95.23 ± 0.13	1.41 ± 0.07	Decrease from ~9.67 to ~8.78 (30 days at 4 ºC)^**^	Decrease from ~9.67 to ~7.17 log CFU/g (gastric digestion)^**^	NI	[113]
Extrusion	*B. infantis* ATCC 15697	Whey protein isolate and sodium alginate	86.15 ± 2.51	1.27 ± 0.12	Decrease from 9.02 ± 0.11 to 7.21 ± 0.04 log CFU/g (28 days at 4 ºC)	33.2% survival (gastric digestion)	Frequency: 800 Hz; Voltage: 800 V; Nozzle aperture: 450 µm	[114]
Extrusion	*B. infantis* ATCC 15697	Pea protein isolate and sodium alginate	90.59 ± 0.46	1.55 ± 0.06	Decrease from 9.81 ± 0.04 to 8.23 ± 0.09 log CFU/g (28 days at 4 ºC)	40.2% survival (gastric digestion) 84.2% survival (intestinal digestion)	Frequency: 800 Hz; Voltage: 800 V; Nozzle aperture: 450 µm	[114]
Extrusion	*B. infantis* ATCC 15697	Whey protein isolate, pea protein isolate, and sodium alginate	94.09 ± 1.76	1.62 ± 0.14	Decrease from 9.89 ± 0.07 to 8.68 ± 0.13 log CFU/g (28 days at 4 ºC)	45.4% survival (gastric digestion) 89.4% survival (intestinal digestion)	Frequency: 800 Hz; Voltage: 800 V; Nozzle aperture: 450 µm	[114]
Nanoemulsion/extrusion	*B. pseudocatenulatum* G7	Sodium alginate and CaCO_3_	ND	729	Decrease from ~8.40 to ~4.51 log CFU/g (4 weeks at 4 ºC)^**^	Decrease from ~9.32 to ~8.70 (exposure to simulated gastric fluid), and to ~6.60 log CFU/g (complete gastrointestinal digestion)^**^	Frequency: 800 Hz; Voltage: 800 V; Nozzle aperture: 200 µm	[112]
Emulsion	*B. bifidum* F-35	Whey protein and sodium alginate	ND	280	Decrease from 10.73 to 9.36 log CFU/g (14 days at 4 ºC)	ND	Organic phase: soybean oil; Cross-linking agent: 10 U of TGase/g	[115]
Emulsion	*B. animalis* BB-12	Sodium alginate	~96^**^	~0.8^**^	Decrease from ~7.87 to ~7.00 log CFU/g (30 days at 4 ºC)^**^	Decrease from ~9.57 to ~8.50/~8.40 (60/120 min in gastric juice), and to ~8.05/~7.58 log CFU/g (50/150 min in intestinal juice)^**^	Organic phase: raspberry oil; Cross-linking agent: 0.1 M CaCl_2_	[116]
Emulsion	*B. animalis* BB-12	Pectin	~95^**^	~2.7^**^	Decrease from ~7.78 to ~7.47 log CFU/g (30 days at 4 ºC)^**^	Decrease from ~9.57 to ~8.41/~8.22 (60/120 min in gastric juice), and to ~7.78/~7.00 log CFU/g (50/150 min in intestinal juice)^**^	Organic phase: raspberry oil; Cross-linking agent: 0.1 M CaCl_2_	[116]
Emulsion	*B. bifidum*, *B. breve*, and *B. longum*	Sodium alginate and chitosan	40.21 ± 3.18	10 to 15	ND	78.28 ± 7.55% survival (sequential exposure to gastric and intestinal juices)	Organic phase: olive oil; Cross-linking agent: 0.2 M CaCl_2_	[117]
Emulsion	*B. bifidum* R0071	Pectin	ND	ND	ND	Decrease from 8.78 ± 0.05 to 8.78 ± 0.06/8.60 ± 0.13 log CFU/g (20/60 min of exposure to gastric juice)	Organic phase: soybean oil; Cross-linking agent: 0.05 M CaCl_2_.2H_2_O	[118]
Emulsion	*B. bifidum* R0071	Sodium alginate	ND	ND	ND	Decrease from 8.74 ± 0.06 to 8.67 ± 0.22/8.53 ± 0.04 log CFU/g (20/60 min of exposure to gastric juice)	Organic phase: soybean oil; Cross-linking agent: 0.05 M CaCl_2_.2H_2_O	[118]
Emulsion	*B. bifidum* R0071	Pectin and osteopontin	ND	ND	ND	Decrease from 8.77 ± 0.02 to 8.79 ± 0.02/8.74 ± 0.08 log CFU/g (20/60 min of exposure to gastric juice)	Organic phase: soybean oil; Cross-linking agent: 0.05 M CaCl_2_.2H_2_O	[118]
Emulsion	*B. bifidum* R0071	Sodium alginate and osteopontin	ND	ND	ND	Decrease from 8.64 ± 0.02 to 8.57 ± 0.06/8.53 ± 0.01 log CFU/g (20/60 min of exposure to gastric juice)	Organic phase: soybean oil; Cross-linking agent: 0.05 M CaCl_2_.2H_2_O	[118]
Emulsion	*B. animalis* F1-7	Sodium alginate	90.67 ± 1.45	297.46	80.43% survival (28 days at 4 ºC)	74.67% survival (gastric digestion)	Organic phase: soybean oil; Cross-linking agent: 0.05-0.8 M CaCl_2_	[119]
Emulsion	*B. animalis* F1-7	Sodium alginate and human milk oligosaccharides	92.19 ± 1.80	308.07	89.50% survival (28 days at 4 ºC)	86.97% survival (gastric digestion)	Organic phase: soybean oil; Cross-linking agent: 0.05-0.8 M CaCl_2_	[119]
Fluid bed	*B. animalis* subsp. *lactis* NH019	Lactose, stearic acid, sodium alginate, hydroxypropyl cellulose, and microcrystalline cellulose	94.86^*^	50 to 300	ND	Decrease of 0.2/0.58 CFU/g (45/60 min in gastric juice)	First coating: 5% (w/w) stearic acid; second coating: 2% (w/w) sodium alginate; Third coating: 5% (w/w) hydroxypropyl cellulose and microcrystalline cellulose	[120]

^*^Calculated from data included in the manuscript; ^**^Approximated from figures included in the manuscript. ND: Not determined; NI: not informed; IAT: inlet air temperature; OAT: outlet air temperature.

### Spray drying

Spray drying is a widely adopted microencapsulation technique with high throughput and cost-effectiveness^[121]^. Fundamentally, the process consists of three stages: atomization of the feed into microscale droplets, rapid moisture removal through exposure to hot drying air, and powder collection via cyclones or separators^[122]^. The high temperatures and air exposure during spray drying impose thermal and oxidative stress on microorganisms. Studies have shown that while spray drying of *B. bifidum* PTCC 1644 can achieve survival rates above 28% with optimized inlet/outlet temperatures and protective excipients, much of the population is typically lost due to heat and oxygen exposure^[123]^. Research highlights that oxygen-sensitive strains may incur membrane and DNA damage when exposed to hot, aerated environments unless specialized formulations are used^[79,124]^. However, recent advances, such as electrostatic spray drying, have demonstrated improved encapsulation efficiencies (over 93%) and therefore survival rates to the drying step, in other *Bifidobacterium* species (i.e., *B. lactis* BL03 and BAL005, *B. bifidum* BB30, *B. longum* BLL2, and *B. infantis* B120) by generating microcapsules with more protective internal structures^[125]^. Furthermore, most bifidobacteria strains microencapsulated by electrostatic spray drying showed significantly higher viability retention (70%-80%) after exposure to gastric fluid in comparison with spray drying (60%-70%) and emulsion (30%-40%). Concurrently, formulation strategies using biopolymer blends like maltodextrin and gum Arabic improve stability during spray drying and aerobic storage^[122]^. For instance, using 90% maltodextrin, 5% isomalto-oligosaccharide syrup, and 5% biosurfactant as a carrier increased the survival of *B. adolescentis* BCRC 14608 during spray drying to 58.1% and to 72% after exposure to pH 3.2 for 3 h (*vs*. 40% for free cells)^[126]^. Although spray drying remains less protective than gentler techniques like freeze drying, its inherent scalability and continuous processing make it well-suited for industrial applications. With precise control of process parameters, such as adequate inlet temperature, rapid drying, and anaerobic pre-conditioning of cultures, spray drying can be optimized to retain viability levels and functional integrity in *Bifidobacterium* spp. formulations intended for poultry^[127]^. Future developments in hybrid spray systems, incorporating oxygen scavengers or encapsulation aids, may further enhance strain-specific preservation strategies. Spray-dried *Bifidobacterium* spp. powders are well-suited for inclusion in feed or drinking water^[128]^. Nonetheless, they are typically not ideal for *in ovo* injection. This method demands high viability and functional integrity, which may be compromised by the thermal and oxidative stress during spray drying.

From an industrialization perspective, spray drying of *Bifidobacterium* spp. offers substantial advantages in terms of scalability and cost-efficiency compared to other drying methodologies. The continuous nature of spray drying enables high-throughput production, reducing labor and energy costs per unit of product, which is critical for commercial viability^[129]^. However, the upfront capital investment for spray drying equipment and the optimization of process parameters can vary depending on strain-specific requirements and formulation complexity, influencing production costs. Economically, studies estimate production costs for spray-dried probiotic powders to be significantly lower than freeze-dried counterparts^[130]^. Furthermore, product stability during downstream feed processing is crucial for ensuring the practical application value of spray-dried *Bifidobacterium* spp. in animal nutrition. Stability tests to 60-100 ºC for different times, conditions that may be present during feed extrusion and pelleting, have shown that appropriately formulated spray-dried powders retain over 70% viability^[131]^. Additionally, the use of protective excipients and post-process coating can further enhance survival rates during storage and incorporation into feed matrices^[132]^. These findings underscore the feasibility of integrating spray-dried *Bifidobacterium* spp. powders into commercial feed production pipelines while maintaining functional probiotic properties. Nevertheless, continuous monitoring and strain-specific optimization remain essential to maximize industrial process efficiency and product stability.

### Freeze drying

Freeze drying, also known as lyophilization, is widely regarded as the gold-standard preservation method for probiotic cultures, particularly for oxygen-sensitive *Bifidobacterium* spp. The process preserves cell viability by avoiding the thermal stress characteristic of other drying methods such as spray drying. Freeze drying involves three key phases: rapid freezing, sublimation, and desorption, which collectively remove water under vacuum at low temperatures (< -40 ºC)^[133]^. Critical to its success is the rapid formation of small, uniform ice crystals during freezing, which minimizes mechanical damage to cell membranes. At the same time, the vacuum conditions protect cells from oxidative damage. The inclusion of cryoprotectants, such as sorbitol, sucrose, trehalose, skim milk, and glycerol, further enhances survival by stabilizing membrane structures and intracellular proteins through hydrogen bonding and vitrification during drying^[134]^. For instance, *B. crudilactis* freeze-dried with sorbitol achieved over 80% initial viability and retained acceptable counts after six months at 4 ºC^[134]^. Similarly, *B. longum* subsp. *longum* Reuter 1963 subjected to optimized freeze drying protocols with trehalose supplementation maintained over 50% viability during storage at 4 ºC with maltodextrin carriers, and cell integrity was largely preserved^[135]^. Moreover, freeze-dried formulations have demonstrated exceptional resistance to simulated gastrointestinal conditions, maintaining over 90% viability after exposure to gastric fluid and bile salts^[136]^.

Despite the superior viability retention offered by freeze drying, its industrial-scale application for *Bifidobacterium* spp. preservation presents challenges primarily related to production costs and process complexity. Freeze drying is inherently resource- and energy-intensive due to the requirement for ultra-low temperatures and extended processing times under vacuum conditions, which translates into higher operational costs compared to other drying methods like spray drying^[137]^. However, recent advances in process optimization and cryoprotectant use, such as trehalose and sorbitol, have enabled more cost-efficient protocols and improved shelf life, thereby reducing losses during storage and transport^[134,135]^. Moreover, economic feasibility studies indicate that although freeze drying has a higher upfront cost, the longer shelf life and improved viability reduce the need for over-formulation, which can offset initial expense in commercial feed applications^[138]^. Furthermore, freeze-dried *Bifidobacterium* formulations are resilient to typical feed manufacturing stresses, such as pelleting and extrusion, provided appropriate protective matrices are included. Oral administration, via incorporation into feed or drinking water, is the most practical and effective route for delivering freeze-dried *Bifidobacterium* spp. in poultry.

### Spray chilling

Spray chilling (also known as spray cooling) is a lipid-based microencapsulation technique that solidifies molten fat, such as cocoa butter or hydrogenated oils, around probiotic cells by atomizing the mixture into a cooled chamber, thereby forming solid microparticles without applying thermal stress^[139]^. This process is particularly advantageous for oxygen-sensitive *Bifidobacterium* spp., such as *B. animalis* subsp. *lactis*, as it avoids the heat and oxidative exposure associated with spray drying, preserving higher viability both immediately after production (higher than 80%-90%) and during refrigerated storage (higher than 50% after 120 days at 4 ºC)^[140]^. Lipid matrices protect anaerobic probiotics by creating an oxygen-impermeable barrier; upon ingestion, these matrices are degraded by digestive lipases, facilitating the gradual release of viable cells into the gut^[141]^. For example, single- and double-layered capsules containing *B. bifidum* BB-12, obtained by using hydrogenated palm oil, presented an encapsulation efficiency of 92% and survival rates of 88% to 75% during gastric and intestinal digestion, respectively^[142]^.

From an industrial perspective, spray chilling is recognized as one of the most cost-effective microencapsulation methods for large-scale production^[143,144]^. It employs relatively inexpensive, widely available lipid materials and simple cooling systems that require less energy compared to many drying methods^[145]^. The process supports continuous production lines with relatively short processing times, which further reduces operational costs^[145,146]^. In practical feed applications, spray-chilled *Bifidobacterium* spp. microcapsules have demonstrated stability against mechanical and thermal stresses typical of feed processing techniques such as pelleting and extrusion. The lipid coating effectively protects the probiotic cells, maintaining significant viability after exposure to such conditions^[147]^. Additionally, these microcapsules retain their functional properties during storage in feed matrices for extended periods, supporting shelf-life requirements necessary for commercial use^[145]^. These findings confirm that spray chilling is a promising method to produce probiotic feed additives compatible with existing industrial feed production workflows. Nonetheless, challenges remain in optimizing encapsulation parameters, such as lipid composition and particle size, to improve release mechanisms and maximize cost-efficiency. Regulatory considerations for lipid excipients in animal nutrition also need to be addressed to facilitate industrial adoption^[143]^.

It is also noteworthy that while spray-chilled *Bifidobacterium* spp. are well-suited for oral delivery through feed or water, the lipid-coated particles are typically too large and may not be compatible with precise injection tools used for *in ovo* delivery^[73,147]^. In conclusion, spray chilling represents a promising, scalable, and economically viable method for formulating anaerobic probiotics like Bifidobacterium spp. for poultry use. Continued research and scale-up trials are essential to optimize microencapsulation parameters and fully unlock its industrial and practical application potential.

### Extrusion

Extrusion encapsulation involves forcing a mixture of microbial cells and hydrophilic polymers, commonly sodium alginate alone or in combination with proteinaceous materials, through a fine nozzle into a Ca^2+^ solution in the form of droplets, inducing rapid gelation and formation of spherical microbeads^[148]^. This gentle, aqueous-based method is particularly advantageous for strictly anaerobic bacteria such as *Bifidobacterium* spp., as it avoids heat and oxygen exposure during processing^[148,149]^. In studies with *B. animalis* subsp. *lactis* BB-12, alginate beads produced via extrusion in combination with glycerol, carrageenan, or inulin yielded high encapsulation efficiency (between 76% and 85%) and sustained viability above 10^6^ CFU/g after 30 days at 4 ºC, indicating robust protection during storage^[150]^. In another study, *B. pseudocatenulatum* G7 co-encapsulated with colloidal antacid CaCO_3_ within calcium alginate microgels to control the internal pH of the beads showed higher storage stability and resistance to gastric and intestinal digestion than free cells [Table 2]^[112]^. Moreover, alginate-ciceritol beads enhanced storage stability after 30 days and *B. animalis* subsp. *animalis* ATCC 25527 viability under gastrointestinal conditions compared with free cells^[113]^. Extrusion enables tailored bead characteristics through adjustments in polymer concentration, nozzle size, and cross-linker levels^[151]^. This control is important to optimize release kinetics, palatability, and feed formulation compatibility. However, the relatively large bead size produced by extrusion limits its use for precise administration methods, such as *in ovo* injection, making oral delivery via feed or drinking water the most practical route for poultry probiotic applications^[73]^.

From an industrial perspective, extrusion microencapsulation is recognized as a cost-effective, scalable, and straightforward method for probiotic microencapsulation. It utilizes readily available, inexpensive materials, such as sodium alginate and Ca^2+^ salts, and employs simple equipment like nozzles and curing baths. This results in comparatively low capital investment and operational costs relative to drying or spray-coating techniques^[152]^. The aqueous and mild processing conditions also reduce energy consumption and eliminate the use of harmful solvents, enhancing cost efficiency and suitability for large-scale production^[88]^.

Extrusion-encapsulated *Bifidobacterium* microbeads demonstrate substantial resilience to temperatures of 60-100 ºC, typical of feed processing, maintaining viability above 70%^[131]^. These microcapsules sustain probiotic functionality and viable counts over several weeks at ambient temperature, supporting satisfactory shelf life for practical commercial use^[131]^. Despite these advantages, further optimization is required to balance bead size, polymer composition, and processing parameters to fine-tune probiotic release profiles, and improve feed palatability with diverse feed formulations. Additionally, regulatory approval of feed-grade biopolymers remains an important step for broader industrial adoption.

In summary, extrusion microencapsulation combines physiological compatibility with industrial feasibility to maintain viability and deliver functional anaerobic *Bifidobacterium* spp. effectively in poultry feed. Ongoing research and scale-up validation will be essential to fully realize the practical and commercial benefits of this technology in the animal feed industry.

### Emulsion

Emulsion encapsulation involves dispersing probiotic-laden aqueous phases into an immiscible oil phase (typically vegetable oil), forming water-in-oil droplets which are stabilized by emulsifiers and subsequently gelled or solidified to create microcapsules^[153]^. Given the inherent thermodynamic instability of conventional emulsions, advanced emulsion techniques, such as nanoemulsions, Pickering emulsions, and Pickering high internal phase emulsions, have been developed to enhance the encapsulation efficiency of probiotics^[154]^. Nanoemulsions are comparatively stable systems characterized by droplet sizes typically smaller than 100 nm^[155]^. In contrast, Pickering emulsions achieve stabilization without the use of traditional emulsifiers; instead, they are stabilized by solid particles, with hydrophobic particles demonstrating greater effectiveness^[156]^. Pickering high internal phase emulsions represent a subset of Pickering emulsions that contain a high fraction of the internal oil phase. By minimizing the exposure of probiotics to water and oxygen, these high internal phase systems exhibit superior encapsulation efficiency and hold significant potential as probiotic delivery vehicles^[157]^. Emulsion encapsulation is especially appropriate for anaerobic bifidobacteria because it enables encapsulation entirely in oxygen-free aqueous environments before emulsification. Moreover, anoxic regions created in the center of the beads protect cells from oxidative damage^[158]^. For instance, *B. bifidum* F-35 was subsequently encapsulated with whey protein and sodium alginate to obtain 280 µm double-layered beads. The microencapsulated bacteria retained significantly higher viability than free cells after 2 weeks at 4 ºC [Table 2], indicating effective protection by the double-layer barrier^[115]^. Similarly, *B. animalis* BB-12 encapsulated within pectin or sodium alginate droplets stabilized by Ca^2+^ and emulsified in rapeseed oil retained over 10^7^ CFU/g of viable cells after 30 days at 4 ºC, and exhibited higher resistance to gastrointestinal conditions than free cells^[116]^. In another study, *B. bifidum* encapsulated by the emulsion technique within resistant starch beads showed better survivability than free and freeze-dried cells after 3-month storage. Moreover, emulsion-encapsulated bacteria presented better resistance to gastrointestinal conditions than free cells^[159]^.

Emulsion parameters, such as water-to-oil ratio, stirring speed, emulsifier type (e.g., lecithin or Tween 80), and droplet size, can be precisely tuned to control bead diameter and release characteristics, facilitating targeted delivery within the host GIT^[160]^. Moreover, the method’s gentle processing, absence of heat, and aqueous formulation render it cost-effective and easily scalable^[161]^.

Recent research highlights the suitability of emulsion microencapsulation for industrial-scale production due to its relative simplicity, scalability, and compatibility with continuous or semi-continuous processing, compared to other microencapsulation techniques^[154,162]^. For instance, millifluidic-assisted and emulsification-based systems can achieve high encapsulation efficiencies (up to 98%), producing microcapsules with optimal size and sphericity for food and feed applications^[162]^. Moreover, employing food-grade polysaccharides and vegetable oils is economically favorable, utilizing inexpensive and readily accessible materials that can be seamlessly incorporated into current production processes^[154]^.

From a stability perspective, emulsion-encapsulated *Bifidobacterium* spp. maintain high viability under feed processing conditions. The use of double-layer encapsulating materials, such as chitosan and sodium alginate, better preserves probiotic viability during storage, ensuring sufficient viable counts for probiotic efficacy at the moment of administration^[162]^.

However, the choice of oil phase and emulsifiers must be compatible with feed formulations and downstream processing, as residual oil may affect palatability or regulatory compliance in feed additives. Therefore, emulsion microencapsulation presents a promising commercial method, provided that process parameters and encapsulating agents are optimized for specific feed matrices and regulatory requirements.

## CONCLUSION

In an era marked by growing restrictions on antibiotic use and increasing demand for sustainable poultry production, *Bifidobacterium* spp. have emerged as promising probiotic candidates capable of enhancing gut health, immunity, and overall performance in poultry. Despite their proven functional benefits, including modulation of the gut microbiota, improvement of intestinal architecture, regulation of immune responses, and inhibition of pathogens, their widespread application in the poultry industry remains limited due to their strict anaerobic nature and sensitivity to environmental stressors. To overcome these challenges, recent advances in administration strategies and microencapsulation technologies have opened new avenues for the practical integration of bifidobacteria into poultry systems. Routes such as *in ovo* injection and cloacal delivery offer high colonization efficiency, while oral and spray-based applications provide scalable and operationally feasible alternatives. Crucially, the use of microencapsulation techniques, ranging from extrusion and emulsion to spray chilling and freeze drying, enhances the survival, stability, and functional performance of bifidobacteria, enabling their deployment even under suboptimal conditions.

Moving forward, the strategic combination of strain selection, encapsulation optimization, and targeted delivery routes holds great potential for fully realizing the benefits of *Bifidobacterium* spp. in poultry health and productivity. Future research should focus on field-scale validations, long-term colonization dynamics, and host-microbe interactions under commercial settings to support the rational design of next-generation probiotic formulations. By aligning biotechnological innovation with practical application, bifidobacteria-based probiotics can become integral components of antibiotic-free and performance-driven poultry production systems.

## DECLARATIONS

### Copyright

© The Author(s) 2025.
